# Challenges in Australian policy processes for disinvestment from existing, ineffective health care practices

**DOI:** 10.1186/1743-8462-4-23

**Published:** 2007-10-31

**Authors:** Adam G Elshaug, Janet E Hiller, Sean R Tunis, John R Moss

**Affiliations:** 1 Discipline of Public Health, The University of Adelaide, Mail Drop 207, Adelaide, SA, Australia, 5005; 2 Hanson Institute, Institute of Medical and Veterinary Science, Adelaide, SA, Australia, 5000; 3 Center for Medical Technology Policy, San Francisco, 4712 Keswick Rd, Baltimore, MD, USA, 21210

## Abstract

**Background:**

Internationally, many health care interventions were diffused prior to the standard use of assessments of safety, effectiveness and cost-effectiveness. Disinvestment from ineffective or inappropriately applied practices is a growing priority for health care systems for reasons of improved quality of care and sustainability of resource allocation. In this paper we examine key challenges for disinvestment from these interventions and explore potential policy-related avenues to advance a disinvestment agenda.

**Results:**

We examine five key challenges in the area of policy driven disinvestment: 1) lack of resources to support disinvestment policy mechanisms; 2) lack of reliable administrative mechanisms to identify and prioritise technologies and/or practices with uncertain clinical and cost-effectiveness; 3) political, clinical and social challenges to removing an established technology or practice; 4) lack of published studies with evidence demonstrating that existing technologies/practices provide little or no benefit (highlighting complexity of design) and; 5) inadequate resources to support a research agenda to advance disinvestment methods. Partnerships are required to involve government, professional colleges and relevant stakeholder groups to put disinvestment on the agenda. Such partnerships could foster awareness raising, collaboration and improved health outcome data generation and reporting. Dedicated funds and distinct processes could be established within the Medical Services Advisory Committee and Pharmaceutical Benefits Advisory Committee to, a) identify technologies and practices for which there is relative uncertainty that could be the basis for disinvestment analysis, and b) conduct disinvestment assessments of selected item(s) to address *existing *practices in an analogous manner to the current focus on *new *and *emerging *technology. Finally, dedicated funding and cross-disciplinary collaboration is necessary to build health services and policy research capacity, with a focus on advancing disinvestment research methodologies and decision support tools.

**Conclusion:**

The potential over-utilisation of less than effective clinical practices and the potential under-utilisation of effective clinical practices not only result in less than optimal care but also fragmented, inefficient and unsustainable resource allocation. Systematic policy approaches to disinvestment will improve equity, efficiency, quality and safety of care, as well as sustainability of resource allocation.

## Background

The term *disinvestment *in health care is gaining prominence internationally. It relates to the processes of (partially or completely) withdrawing health resources from any existing health care practices, procedures, technologies or pharmaceuticals that are deemed to deliver little or no health gain for their cost, and thus are not efficient health resource allocations. The goal of reducing the use of ineffective technologies or practices has been central to Evidence-Based Medicine (EBM) for well over a decade. In the early 1990s claims were made that in all areas of health care, "30–40% of patients do not receive treatments of proven effectiveness" [1], and, "20–25% of patients have treatments that are unnecessary or potentially harmful" [2]. Since then, advances have been made in Australia, and internationally, to improve primarily the safety of health care, but also clinical and cost-effectiveness. Improvements have been achieved through the collaborative work of national and regional health departments, health care institutions, professional colleges, academia and numerous organisations. In Australia these include but are not limited to the National Institute of Clinical Studies (NICS), the Australian Commission on Safety and Quality in Health Care – formerly the Australian Council for Safety and Quality in Health Care, the Australasian Association for Quality in Health Care (AAQHC), Effective Healthcare Australia (EHA), and the National Health and Medical Research Council (NHMRC). Furthermore, health technology assessment consultancy groups such as Adelaide Health Technology Assessment (AHTA) are increasingly involved in supporting current policy processes.

Considerable effort and resources have been invested in Australia in developing well-defined criteria and evidence-based policy processes for assessing *new *and *emerging *health technologies, surgical procedures and pharmaceuticals to gauge their safety, effectiveness and cost-effectiveness [3,4]. Reimbursement approval (and therefore universal access through Australia's Medicare system) for these new services, as well as the withdrawal of reimbursement for existing services, rests with the Australian Government Minister for Health and Ageing under advice from the Medical Services Advisory Committee (MSAC) and, for pharmaceuticals, the Pharmaceutical Benefits Advisory Committee (PBAC). The MSAC and the PBAC employ stringent review processes based on the existence of quality data and evidence that are available at the time of assessment. Underpinning the disinvestment movement, however, is a recognition that these stringent assessment methods are relatively novel, and that the processes to date have focused overwhelmingly on new and emerging practices, technologies and pharmaceuticals and not on existing services (even though this is within the mandate of the MSAC). Australia therefore, like other countries, suffers from a legacy whereby many currently implemented health care interventions were in use prior to well-defined standards of cost-effectiveness becoming a criterion for reimbursement. Thus there is concern that health services of limited effectiveness may still be in practice nation-wide. The Chief Executive Officer of Australia's National Institute of Clinical Studies (NICS) has stated,

*We do not know how much of the total healthcare Australians receive is based on the best available evidence; studies of a number of specific conditions show that there are gaps between what is known and what happens in practice *[5].

While processes such as clinical practice guidelines development and implementation continue to tackle aspects of this problem, disinvestment focuses on a complementary but parallel facet by examining practices that should be reduced or in some cases eliminated completely. We classify the principal challenges for disinvestment as follows:

1) Lack of dedicated resources by key stakeholders to build and support disinvestment policy mechanisms

2) Lack of reliable administrative mechanisms to identify and prioritise technologies and/or practices with relative uncertainty as to their clinical and cost-effectiveness

3) Political, clinical and social challenges to removing an established technology (including challenges to limiting coverage to specific patients, institutions, or providers)

4) Lack of published studies that clearly demonstrate that existing technologies/practices provide little or no benefit

5) Inadequate resources to support a research agenda to advance disinvestment methods

## Discussion

The following discussion will examine some of the elements involved in addressing these challenges. The discussion will highlight what is occurring currently with implications for what ought to occur in order to support effective disinvestment. We will present two brief case studies to illustrate several complexities with accompanying recommendations.

1) Lack of dedicated resources by key stakeholders to build and support disinvestment policy mechanisms

The bearer of financial risk for the cost of healthcare perhaps has the greatest incentive to drive a disinvestment agenda. In the USA this might include public and private payers, purchasers or large employers. Whereas in government managed or mixed-model health care systems it could be the national insurer and/or the private health insurance industry. In Australia, the MSAC faces issues in its current policy processes and capacity to support effective disinvestment. While for new devices or pharmaceuticals the burden for proving effectiveness lies with the sponsor, for potentially obsolete technology or practices, the opposite occurs. The regulator or payer (the Australian government with advice from the MSAC) firstly has to identify or be made aware of a doubtful practice, to commission reviews, and then to mount a compelling argument for ineffectiveness and/or cost-ineffectiveness. For illustrative purposes this may be described as somewhat analogous to standards of law. That is, adding an item to the schedule of benefits (or its equivalent in international terms) is beholden to a 'balance of probabilities' standard whereas removal of an item requires a standard of evidence that is 'beyond reasonable doubt'. As will be discussed further, this identification and appraisal process is in itself complex, but even if it were not, the MSAC has a full agenda with applications for new and emerging technologies and hence has limited capacity to address existing services. This is evident in communications with the MSAC members that highlight their workload, and the focus of that work. At a recent meeting of the MSAC, the 22 committee members were faced with 700 pages of documentation to consider. All of that material was for *new *and *emerging *technologies and practices; none was for *existing*, potentially ineffective health care. In Australia we therefore appear to be 'stuck with the old and overwhelmed by the new'. Irrespective of the successes the Australian policy model has had in assessing new and emerging technologies, there appears to be a lack of capacity to address both new and existing practices. Or arguably the capacity exists but is not being appropriately harnessed at present, which may reflect a lack of political will. In any case disinvestment is limited. This limitation in capacity may be, in part, a result of the political and professional complexities associated with disinvesting existing practices (discussed further below). It also points to the growing need for a political paradigm shift in order to foster policy-driven disinvestment capacity.

2) Lack of reliable administrative mechanisms to identify and prioritise technologies and practices for which there is relative uncertainty as to clinical and cost-effectiveness

Disinvestment may be easier with pharmaceuticals and/or when adverse events occur. The process is more complex when individual are not harmed by existing practices but over-treated or ineffectively treated. That is, subjected to diagnosis or treatment that is safe but of little or no meaningful clinical benefit (i.e. supported by the existence of compelling clinical- and cost-effectiveness evidence). A register for 'ineffectively-treated' does not exist in the same way as an adverse event register exists for pharmaceuticals, or an adverse event register within tertiary care settings. Further compounding this issue is the limited number of groups in Australia (or indeed elsewhere) with a clear directive and sufficient resources or incentive to seek out, identify and investigate potentially redundant/ineffective procedures. Wilensky has recently intimated that similar limitations (and potential for improvement) exist in the United States of America (USA) [6]. This reflects a view that substantial additional investment is required to support evidence-based review of not only new and emerging but also existing health care technologies, including comparative studies examining new versus existing practices.

In Australia the incentive pendulum supports diffusion and not retraction or 'disinvestment'. The current MSAC model appears geared (and effectively so) toward controlling the tap as it is turned on, not toward neutralizing the flow through active disinvestment. It is interesting for example, that old technologies or practices are not formally de-commissioned as new items are approved. Instead, the range of options grows ever larger. And although some (including at least one of the current authors) purport that many practices simply fade away or die a slow death, the question remains whether this represents a sound policy approach to resource allocation and clinical excellence in health care.

Currently the MSAC provides advice on whether a proposed new service is as or more safe, effective and cost-effective than a comparator. There is nothing to stop the MSAC from recommending the removal of one service as it recommends addition of another service to the reimbursement schedule should the new service have demonstrably better cost effectiveness for a given indication. That the comparator is not automatically removed from the schedule highlights a challenging issue for a disinvestment programme. The new service (even if superior) may take time to diffuse into practice and become accepted by the medical profession. Premature disinvestment of the comparator may disadvantage patients where the new service was not yet available and thereby raise issues of access and equity. The evidence supporting the new technology may not go as far as assessing the cost-effectiveness of disinvestment of its comparator (in terms of capital investment, training, and changes to clinical workflow). Decision-making in disinvestment must take account of these factors.

3) Political, clinical and social challenges to removing an established technology (including challenges to limiting coverage to specific patients, institutions, or providers)

For existing technologies or practices there are complexities that do not beset those that are new or emerging. These relate to their entrenched status and include for example:

• Resistance to change due to established clinical training and practice paradigms

• Multiple clinical, consumer and political interests

• Clinical and consumer influence and preferences, and supplier-induced demand

• Incentive and disincentive mechanisms

• The sunk costs of human and physical capital which would thereby become obsolete

Schon describes how social systems work hard to resist change, a phenomenon he labels 'dynamic conservatism' [7]. Research and applied decision making in this area must therefore include analysis of the evidence for safety, effectiveness and cost-effectiveness as well as social, ethical and political analyses to explain why ineffective health care practices persist. Only then is there scope to address how ineffective practices can be disinvested.

For the clinician there is often concern that disinvestment represents a blunt instrument of rationing, one that may restrict clinical autonomy and reduce patient choice. But can continued investment in health care occur without thoughtful, measured disinvestment? There is an economic imperative to do so for the sake of sustainability. There is also an ethical imperative for the delivery of quality health care and a best practice imperative for clinical purchasers and providers. Disinvestment will free up resources for those practices that have demonstrated effectiveness. Furthermore, disinvestment should not be seen as an all or nothing approach. Removing a reimbursement item number from the Schedule of Medical Benefits (or the equivalent action in international terms) would be an extreme example of successful disinvestment. There may well be a policy-guided process of measured retraction including restricting the indications for particular services.

Important in any disinvestment policy model is recognition and consideration of perceived threats that may be raised by disinvestment. We have included a case study involving Assisted Reproductive Technologies (ART) for women over the age of 42 years to explore some potential threats and argue that these are legitimate considerations in a disinvestment analysis (See Table 1).

**Table 1:** Case Study 1 Assisted Reproductive Technologies (ART) for women over 42 years of age

ART does not specifically affect morbidity or mortality (with some exceptions). The debate has, and continues, to occur prominently in the broad community as well as government. In 2003, age-specific success rates of In Vitro Fertilisation (IVF) cycles using fresh oocytes were [28]: • 27.7% for women aged 25–29 • 24.9% for women aged 30–34 • 17.1% for women aged 35–39 • 6.8% for women aged 40–44 • 2% for women aged ≥ 42 (some clinics have cited success rates of 5–10%) This success rate for women aged 42 years or more has been cited by one Australian clinic as a reason for refusing to treat women of that age [29]. Politicians have also engaged with this debate. In April 2005 the Australian Government Minister for Health and Ageing considered limiting ART funding under Medicare to three cycles per year for women 42 and under, and to three cycles in total for women over this age [30]. Rising expenditure on ART and the low age-specific success rates were cited as justification for this policy. This proposal for reduced support was subsequently abandoned by the Australian Prime Minister in May 2005. Table 3 further explores some of the methodological issues associated with this as a potential case study in disinvestment.

There is clear evidence of limited effectiveness for ART with increasing maternal age. There are also social, political and ethical considerations that any disinvestment strategy (and methodological framework) must take into consideration. The complexities involved in any potential disinvestment analysis of this issue support the need for methodological advances in this area of health services research. Such advances are required if health services such as ART are to be moved out of the 'too hard basket' and into active assessment, debate and appraisal.

Another barrier to disinvestment is the notion that medical technology has provided good value for money over time, and that regulators' efforts to restrict coverage (or disinvest) may reduce incentives for investment and innovation, thereby impeding the future flow of valuable technologies [8]. We may need to tolerate a certain level of payment for low value or relatively ineffective technologies and practices because such is the market environment that makes possible the valuable medical breakthroughs.

4) Lack of published studies that clearly demonstrate that existing technologies/practices provide little or no benefit

For many technologies and practices there is evidence supporting varying degrees of effectiveness when used in certain contexts (for example to certain patient groups with varying degrees of predictive prognoses). However, there are also examples of inappropriate application of otherwise effective technologies or procedures, culminating in ineffective care and inefficient resource allocation. Notable examples of this are presented in the work of Wennberg, disseminated in the Dartmouth Atlas highlighting geographic variation in the use of a range of procedures [9]. Under such conditions a degree of measured retraction of practice is desirable, with resultant disinvestment. Clinical practice modification and refinement techniques, perhaps via clinical practice guideline development and implementation, have demonstrated efficacy. As noted by Miles and co workers,

*clinical practice guidelines remain, when certain conditions are met and their limitations fully understood, useful vehicles for implementing agreed changes to clinical practice and service provision. Certainly, the process of deriving and implementing clinical practice guidelines has developed into a science in its own *[10].

However, for some technologies or practices the evidence for effectiveness is either less clear, or is negative, yet the practice persists. In these instances, partial or complete removal (from funding) may be necessary. Substantial challenges exist, particularly around adequate and timely definition and acceptable proof of inferiority. This is not only conceptually difficult but also limited by data availability and interpretation. Further complicating this is the lag that often exists in the reliable reporting of health outcomes data based on clinical practice. Table 2 (Case Study 2) presents a case highlighting potential complexities of 'evidence' in disinvestment review decisions. These, together with considerations from the ART example, are further addressed in Table 3.

**Table 2:** Case Study 2 Upper airway surgical procedures for adult Obstructive Sleep Apnea (OSA)

Approximately one in five adults has at least mild OSA and one in 15 adults has OSA of moderate or worse severity [31]. The condition is an independent risk factor for substantial morbidity(ies) with implications also for mortality [32]. Currently, upper airway surgery is a second-line treatment alternative to an established non-surgical gold-standard. A recent meta-analysis of these surgical procedures reported success rates at [33]: • 13% for Phase I procedures (including uvulopalatopharyngoplasty [UPPP], laser-assisted uvulopalatoplasty [LAUP], hyoid suspension [HS], genioglossus advancement [GA], and radiofrequency volume reduction of soft tissue [RFVR]) • 43% for advanced Phase II procedures (maxilla and/or mandible advancement (MMA) requiring up to three days Intensive Care Unit recovery). • Two reports of patient satisfaction highlight that surgery has a high postoperative morbidity rate, a high patient-reported failure rate and a low level of satisfaction with 53% [34] and 61% [35] patient-reported 'regret' rates. • The Cochrane review in this area [36] supports the restricted use of these operations and yet Australian Medicare data highlights that these procedures are widespread and increasing. Despite the existence of these procedures for over a decade, debate regarding their efficacy has recently intensified, as to whether these success rates presented above represent 'highly effective treatment', sufficient enough to confer improved health outcomes [37-39]. Disagreement has occurred primarily between relevant medical specialties (i.e. surgeons and sleep medicine physicians). Importantly, there has been a lag in presentation of the necessary evidence to inform and advance such a debate, principally as no policy group has been assigned a stake in the collection or generation of such evidence, hereto it has accrued via the noble but *ad hoc *actions of clinical groups.

**Table 3:** Investigative issues associated with the chosen health care practices

**Health Care Technology/Practice**	**Setting**	Interest from methodological and policy perspectives*	**Key Issues**
ART ≥ 42 years of age	Clinic or Hospital	Harmful x Clinically Effective x Cost Effective ? Appropriate ? Socially Valued √ Universally Accessible ?/x Ethical ?	- Marginal clinical and cost-effectiveness (on population basis) but limiting its use poses problems: it is highly beneficial from perspective of concerned individuals - Therapy has equivocal purpose - Highly valued by recipients and potentially by society broadly - Ability to pay: user vs society - Equity of access - Medical vs social infertility - Opportunity cost
Upper airway surgery for adult OSA	Surgical Theatre	Harmful ? Clinically Effective ?/x Effective Alternative √ Cost Effective x Appropriate ? Necessary ? Socially Valued ?/√ Ethical ?	- Limiting its use should not, in theory, pose any problems, but pressures are strong from clinical interest groups - Complex practice paradigms/incentives - Small, homogeneous craft group - Equipoise/clinical uncertainty - Perspectives of patients who value the potential of a surgical 'fix' - Is this preference based on sound evidence or supplier induced demand? - Opportunity cost

*Key: ? = Unsure or in question; x = Limited or evidence in the negative; √ = Evidence in the positive

Within this context there is scope for expanding the capacity to conduct primary and secondary research of stand alone and comparative effectiveness for existing as well as new technologies [11,12]. The disinvestment considerations highlighted in Table 3 require methodological advances but also time for thorough and rigorous review. In such cases there may be potential for a 'funding with evidence generation' mechanism (also called 'only in research' in the United Kingdom (UK) and 'coverage with evidence development' in the USA). This approach is currently applied for some emerging technologies but could be adapted for existing practices. Here, payers/regulators may allow ongoing funding only for a defined period of time to allow for the generation and/or analyses of necessary evidence. Currently there is provision for use of this approach with new technologies by the MSAC.

5) Inadequate resources to support a research agenda to advance disinvestment policies and methods

The discussion thus far highlights the need for methodological advances to support disinvestment decision making. In Australia, health technology assessment groups conduct and present the synthesised, evidence-based reviews that support the MSAC and the PBAC reimbursement decisions for new and emerging technologies and pharmaceuticals. Health Technology Assessment (HTA) incorporates multidisciplinary fields of policy analysis, and has broadened, "*from primarily addressing effectiveness and safety issues to covering a broader array of issues such as psychological, organizational, ethical and legal aspects*" [13]. HTA studies the medical, social, ethical, and economic implications of development, diffusion, and use of health technologies, practices and services. HTA agencies together with health services and policy researchers generally, are well positioned to take a lead role in supporting the disinvestment of existing, ineffective health care practices. To do this effectively requires collaborative research and recently announced funding increases from the National Health and Medical Research Council (NHMRC) for Health Services Research offers potential here. But in Australia HTA groups largely operate as contract research organisations and with relatively short-term contracts these groups tend to lack capacity to build or support a broad methodological research agenda for the disinvestment of existing, ineffective health care practices. This phenomenon is not restricted to Australia, and as Lehoux has observed, the involvement of academic institutions in HTA has the potential to bring with it conflicting legitimacies between the production of traditionally scientific versus user-oriented knowledge [8]. With concerted capacity building and research initiatives aimed at improving linkage and exchange between policy advisors/makers and academic researchers (HTA specialists), priority driven, contextually relevant research would be facilitated and could support decision-making processes that policy makers need. Moreover, it would contribute much-needed methodological advances and align with the four main characteristics of action research defined by Hart and Bond [14]: (1) collaboration between researchers and practitioners; (2) solution of practical problems; (3) change in practice; and (4) development of theory.

Collaborative models amenable to disinvestment strategies are increasingly being developed and adopted internationally, with some based on the priority setting developments described by Mooney [15,16]). The strategic 'Linkage and Exchange' and 'Participatory Action Research' programs in Canada [17-21] deal with similar issues. Recently, the UK's National Institute for Clinical Excellence (NICE) announced a formal policy agenda to *"purge from the NHS treatments that do not improve health or are poor value for money" *[22]. It is interesting to note however that subsequent to the NICE disinvestment agenda being released, a formal UK Treasury report into UK health research and funding highlighted the challenges faced:

*The delivery of robust scientific appraisal for technologies is coming under increasing challenge as a result of its reliance on methodologies that, it is widely recognized, need further development, given that Health Technology Assessment (HTA) is a relatively new science. Appropriate research is required to address these challenges. In particular, research into methodology for... disinvestment methods *([23], page 103)

In the United States the 'Developing Evidence to Inform Decisions about Effectiveness' (DeCIDE) Research Network has been implemented to support the development of new scientific knowledge through research on the outcomes of health care items and services. This is a clear policy directive in line with Section 1013 of the Medicare Modernization Act of 2003. Wilensky has commented that a window of opportunity currently exists in the USA for the development of a Center for Comparative Effectiveness, particularly to address pharmaceuticals [6]. We believe the greater challenge is to incorporate the assessment of *existing *health care practices and technologies, as well as pharmaceuticals. Fundamental to this is an expanded process of medico-vigilance – monitoring of whether the practice/technology is not only safe but effective and cost-effective in 'real' use outside the tightly controlled environments in which initial evidence may have been collected. Beyond this the social, ethical and political complexities must be accounted for as explicit components of any disinvestment analysis.

Based on this discussion we present the following recommendations of initiatives to be implemented in Australia (and internationally where appropriate):

♣ Government partnerships to involve the professional colleges and relevant stakeholder groups (consumer/community) to put disinvestment on the agenda including awareness, collaboration and improved health outcome data generation and reporting (ongoing medico-vigilance).

♣ Dedicated funds and distinct processes (i.e. transparent legal framework) within the MSAC and where appropriate the PBAC to:

○ Identify technologies and practices for which there is relative uncertainty for disinvestment analysis/review

○ Conduct disinvestment assessments/reviews of the selected item(s)

This should involve an expanded capacity for these committees (or others adopting a similar model) to address existing practices in an analogous manner to their current focus on new and emerging technologies, practices and pharmaceuticals.

○ At this juncture in Australia's health policy landscape, collaborative links to advance disinvestment should be made between the relevant stakeholder bodies, including: the MSAC/PBAC, state departments of health, the Australian Commission on Safety and Quality in Health Care, the National Institute of Clinical Studies (and the NHMRC more broadly).

♣ For existing health care services for which there is relative uncertainty, consideration for the implementation of 'funding with evidence generation'. That is, ongoing reimbursement only agreed for a limited number of years pending evidence generation/review processes – with the possibility of extensions being considered.

♣ Dedicated funding and cross-disciplinary collaboration to build health services and policy research capacity with a focus on advancing disinvestment research methodologies and decision support tools for policy stakeholders.

Disinvestment from existing health care practices that offer little or no health gain is a policy challenge that requires greater attention, both for quality of care and sustainable resource allocation. Disinvestment may well depend less on the availability of resources than on the political will to support work in this area.

## Summary

Australia currently has limited systems in place to support the disinvestment of currently used ineffective, or inappropriately applied, health care practices. With the growing burden of chronic health conditions, addressing this limitation should be recognised as an emerging national priority area [24]. This discussion piece is intended to stimulate further debate in this area (see also [25-27]). The potential over-utilisation of less than effective clinical practices and the potential under-utilisation of effective clinical practices not only result in less than optimal care but also fragmented, inefficient and unsustainable resource allocation. Systematic policy approaches to disinvestment will improve equity, efficiency, quality and safety of care, as well as sustainability of resource allocation. Developing health services and policy research methodologies that tackle these complexities to assist policy-makers will advance the disinvestment agenda. This is a growing area of priority setting in health care that requires national and international perspectives, debate and collaboration.

## Competing interests

Adam Elshaug received funding for this project from the Faculty of Health Sciences of the University of Adelaide. Professor Hiller is director of Adelaide Health Technology Assessment (AHTA). This organisation is contracted to complete evaluations of health technologies. Assoc Prof Moss provides health technology assessments to the Australian Government Department of Health and Ageing as a consultant. Dr Tunis is the Founder and Director of the Center for Medical Technology Policy in San Francisco, where he consults with health care decision makers and stakeholders to support the rapid evaluation and effective use of new medical technologies. In all other respects the authors declare they have no competing interests.

## Authors' contributions

AE conceptualised this paper, completed the first substantive draft and contributed to subsequent draft revisions. JH and JM contributed substantively to subsequent draft revisions. ST contributed an international perspective (primarily USA) and substantive editorial comments on two drafts. All authors read and approved the final manuscript.
